# Progression of diabetic nephropathy and vitamin D serum levels: A pooled analysis of 7722 patients

**DOI:** 10.1002/edm2.453

**Published:** 2023-09-24

**Authors:** Yomna E. Dean, Sameh Samir Elawady, Wangpan Shi, Ahmed A. Salem, Arinnan Chotwatanapong, Haya Ashraf, Tharun Reddi, Prashant Obed Reddy Dundi, Waleed Yasser Habash, Mohamed Yasser Habash, Safaa Ahmed, Hana M. Samir, Ahmed Elsayed, Aryan Arora, Abhinav Arora, Abdelrahman Elsayed, Tamer Mady, Yousef Tanas, Yusef Hazimeh, Mohamed Alazmy, Hani Aiash

**Affiliations:** ^1^ Faculty of Medicine Alexandria University Alexandria Egypt; ^2^ Neuro‐endovascular Surgery Department Medical University of South Carolina Charleston South Carolina USA; ^3^ Faculty of Medicine Chulalongkorn University Bangkok Thailand; ^4^ Faculty of Medicine Suez Canal University Ismailia Egypt; ^5^ Faculty of Dentistry Chulalongkorn University Bangkok Thailand; ^6^ Arkansas College of Osteopathic Medicine Fort Smith Arkansas USA; ^7^ Karnataka Institute of Medical Sciences Rajiv Gandhi University of Health Sciences Bengaluru India; ^8^ New Giza University Giza Egypt; ^9^ Faculty of Medicine Kasr Al‐Ainy, Cairo University Cairo Egypt; ^10^ Syracuse University Syracuse New York USA; ^11^ College of Medicine Royal College of Surgeons Dublin Ireland; ^12^ International American University, College of Medicine Vieux Fort Saint Lucia; ^13^ Lebanese University Beirut Lebanon; ^14^ Zahraa Hospital, University Medical Center Beirut Lebanon; ^15^ Medical director, Dhaman Health Assurance Company Kuwait Kuwait; ^16^ SUNY Upstate Medical University Syracuse New York USA

**Keywords:** albuminuria, diabetic nephropathy, vitamin D

## Abstract

**Background and Aim:**

Low serum Vitamin D levels have been associated with diabetic nephropathy (DN). Our study aimed to analyse the serum levels of vitamin D in patients suffering from DN and the subsequent changes in serum vitamin D levels as the disease progresses.

**Methods:**

PubMed, Embase, SCOPUS and Web of Science were searched using keywords such as ‘25 hydroxyvitamin D’ and ‘diabetic nephropathy’. We included observational studies that reported the association between the serum 25 hydroxy vitamin D levels and diabetic nephropathy without restriction to age, gender, and location. R Version 4.1.2 was used to perform the meta‐analysis. The continuous outcomes were represented as mean difference (MD) and standard deviation (SD) and dichotomous outcomes as risk ratios (RR) with their 95% confidence interval (CI).

**Results:**

Twenty‐three studies were included in our analysis with 7722 patients. Our analysis revealed that vitamin D was significantly lower in diabetic patients with nephropathy than those without nephropathy (MD: −4.32, 95% CI: 7.91–0.74, *p*‐value = .0228). On comparing diabetic patients suffering from normoalbuminuria, microalbuminuria, or macroalbuminuria, we found a significant difference in serum vitamin D levels across different groups. Normoalbuminuria versus microalbuminuria showed a MD of −1.69 (95% CI: −2.28 to −1.10, *p*‐value = .0002), while microalbuminuria versus macroalbuminuria showed a MD of (3.75, 95% CI: 1.43–6.06, *p*‐value = .0058), proving that serum vitamin D levels keep declining as the disease progresses. Notwithstanding, we detected an insignificant association between Grade 4 and Grade 5 DN (MD: 2.29, 95% CI: −2.69–7.28, *p*‐value = .1862).

**Conclusion:**

Serum Vitamin D levels are lower among DN patients and keep declining as the disease progresses, suggesting its potential benefit as a prognostic marker. However, on reaching the macroalbuminuria stage (Grades 4 and 5), vitamin D is no longer a discriminating factor.

## Graphical abstract

Serum vitamin D levels are lower among diabetic nephropathy (DN) patients compared to diabetics patients not suffering from DN. Serum vitamin D levels further declined as the DN progressed from microalbuminuria to macroalbuminuria. Serum vitamin D levels did not significantly differ among Grade 4 and Grade 5 DN.

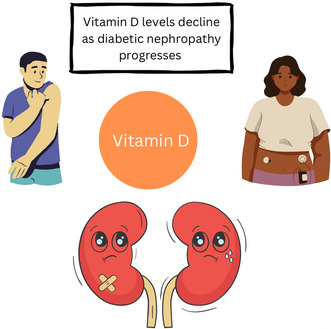

## 1 INTRODUCTION

Diabetic nephropathy (DN), which also falls under the umbrella term Diabetic Kidney Disease (DKD), is a severe microvascular complication of diabetes representing one of the leading causes of end‐stage renal disease worldwide and a significant cause of morbidity and mortality in both Type 1 and Type 2 diabetes. Approximately one‐third of diabetic patients develop diabetic nephropathy.^1^ The diagnostic criteria for diabetic nephropathy are elevated blood pressure, progressive decline in glomerular filtration rate (GFR) and Persistent albuminuria (more than 300 mg/day) on at least two visits 3–6 months apart.^2^ The widely used clinical staging system by Mogensen divides diabetic nephropathy into five stages, including hyperfiltration, silent stage, incipient nephropathy, overt nephropathy and renal failure stage.^3^

The exact mechanism of diabetic nephropathy is still unknown, but it seems to result from the interaction between genetic susceptibility and environmental factors, mainly metabolic in origin. This interaction leads to structural changes in glomerular capillaries and renal tubules, including glomerular basement membrane thickening, mesangial expansion, thickening of the glomerular basement membrane, effacement of podocytes foot process, decreases in number and density of podocytes, and tubulointerstitial fibrosis. Identifying the important indicators significantly associated with DKD plays a pivotal role in understanding the underlying mechanism; these include urine proteins, peptides, markers of Inflammation and oxidative stress, and exosome markers. The urine protein markers worth highlighting are urinary liver fatty acid‐binding protein (L‐FABP), neutrophil gelatinase‐associated lipocalin (NGAL), N‐acetyl‐β‐D‐glucosaminidase (NAG) and Kidney injury molecule‐1 (KIM‐1) along with Vitamin‐D binding proteins. Collagen fragments are an important peptide biomarker, while TNFR levels are one of the chief biomarkers of inflammation and oxidative stress. Copeptin (a surrogate marker of arginine vasopressin) is also significantly associated with DN. Regarding the exosome markers, there are three important proteins that are present in patients with DKD: alpha‐microglobulin/bikunin precursor (AMBP), histone‐lysine N‐methyltransferase (MLL3) and voltage‐dependent anion‐selective channel protein 1 (VDAC1).^4^, ^5^

The role of Vitamin D extends beyond the regulation of calcium and phosphate levels; mice that lack vitamin D receptors (VDR) tend to develop a more severe form of DN.^6^ A possible explanation is that vitamin D is an inhibitor of the renin‐angiotensin system (RAS); Angiotensin II (AT II) has been shown to increase the expression of podocyte nephrin protein, which plays a role in the development of proteinuria. Additionally, AT II increases the apoptosis of podocytes, which further contributes to proteinuria and mesangial expansion Via increased expression of transforming growth factor‐beta (TGF‐β). Vitamin D inhibits the pro‐fibrotic growth factors; VDR‐negative mice had increased mesangial expansion and were more susceptible to renal injury.^6^, ^7^ A study conducted by Yang et al.^8^ also gives a deeper understanding of the role Vitamin D has to play in the occurrence of Diabetic Nephropathy. It demonstrated how Vitamin D can partially reverse the proinflammatory effects of lipopolysaccharide (LPS) and interleukin‐15 (IL‐15) which induced the alteration of actin skeleton in THP‐1 cells, activated STAT5 signalling pathway, increased the release of interleukin‐6 (IL‐6) and monocyte chemoattractant protein‐1 (MCP‐1), and triggered an inflammatory response.

This study aimed to analyse the association between the serum level of vitamin D and the progression of DN and its potential use as a prognostic marker.

## 2 METHODS

### 2.1 Search strategy

A literature search of the following databases (PubMed, Scopus, and Web of Science) on 15 May 2022, using key terms on title, abstract, and MESH terms such as ‘vitamin D’, ‘Calcidiol’, ‘25‐hydroxycholecalciferol’, ‘diabetic nephropathy’, ‘diabetic renal’ and ‘diabetic kidney’, ‘diabetes renal’ was performed to identify studies of interest.

### 2.2 Inclusion and exclusion criteria

We screened studies by titles and abstracts according to the following criteria:

*Inclusion criteria*: observational studies (cross‐sectional and case‐control) reporting the association between the serum level of 25 (OH) D (25‐hydroxy vitamin D) and diabetic nephropathy without restriction to age, gender, location, or language, but we included studies conducted on humans only.

*Exclusion criteria*: Editorials, letters to the editor, commentaries, reviews, systematic reviews, meta‐analyses, case reports, case series, animal studies and studies where diabetic patients received vitamin D supplements; in case of duplicate studies, the most recent study with the most extensive study population was included.

### 2.3 Study selection

Two independent reviewers (W.S. and A. C.) screened the studies according to our criteria. A third independent reviewer (T.R.) was assigned to resolve the conflict if they did not achieve a consensus.

### 2.4 Data extraction and quality assessment

Two independent extractors (H.A. and A.E.) extracted the data, and if they did not reach an agreement, a third extractor (S.A.) resolved the conflict.

For the baseline and summary, the following data were extracted from the eligible studies: the first author of the study, year of publication, study design, number of participants, age of participants, sex of participants, average BMI, Vitamin D assay and Vitamin D deficiency cut‐off value.

For the outcomes, the following data were extracted: Vitamin D levels from diabetic patients not suffering from nephropathy, diabetic patients suffering from nephropathy, and diabetic Patients with normoalbuminuria, microalbuminuria, macroalbuminuria and different diabetic nephropathy grades.

The risk of bias was assessed utilizing Newcastle‐Ottawa Scale (NOS) items,^9^ with a nine‐point score, to evaluate the quality of observational studies. We defined the observational studies with an NOS score of ≥7 stars as high quality and an NOS score of <7 stars as low quality.

## 3 RESULTS

### 3.1 Literature review

**FIGURE 1:**
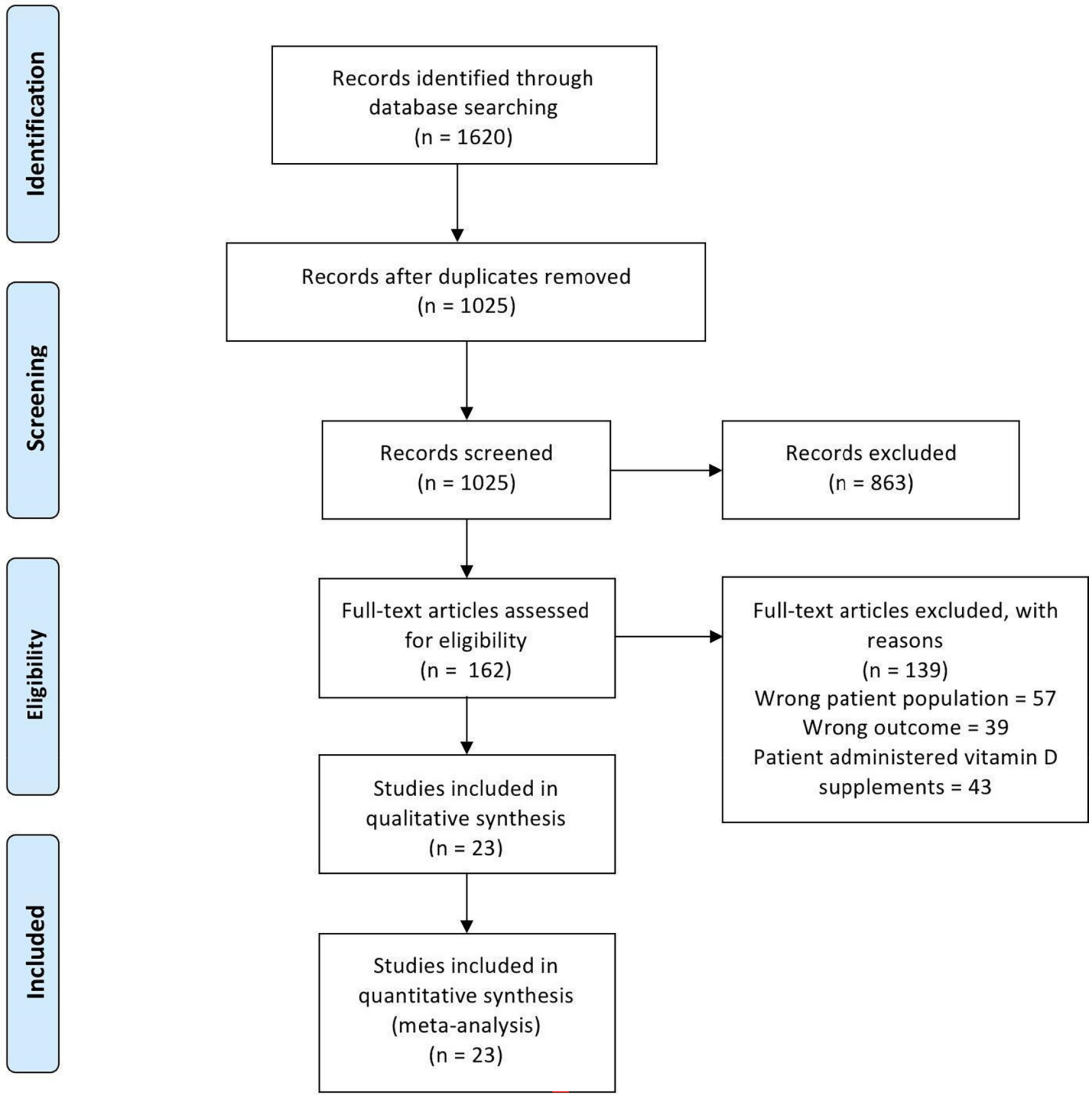
PRISMA flow diagram.

A search of the electronic databases yielded 1620 articles. After removing 595 duplicate articles, 1025 remained. Through screening titles and abstracts, 863 articles were excluded, and 162 papers were assessed for consideration of inclusion and exclusion criteria. After a full‐text assessment for eligibility, 141 articles were excluded. Ultimately, 23 articles were included in the meta‐analysis,^10^, ^11^, ^12^, ^13^, ^14^, ^15^, ^16^, ^17^, ^18^, ^19^, ^20^, ^21^, ^22^, ^23^, ^24^, ^25^, ^26^, ^27^, ^28^, ^29^, ^30^, ^31^, ^32^ as shown in the PRISMA^33^ (Figure 1).

### 3.2 Characteristics of included studies

The combined data set of all studies in our meta‐analysis gathered 7722 patients. The baseline characteristics of the patients included in our study are summarized in Table 1. The quality assessment of included studies using NOS for case‐control studies and a modified version for cross‐sectional studies is summarized in Tables S1 and S2, respectively.

**TABLE 1:** Baseline characteristics of the included studies.

Author year	Country	Study design	Sample size	Age, mean (SD	Male (%)	BMI, mean (SD)	Sample type	Vitamin D deficiency cut‐off (ng/ml)	Vitamin D assay
Abdella^10^	Kuwait	Cross‐sectional	309	44.57 ± 17.92	40		Serum	≤50	Radioimmunoassay on Cobas e411
Bajaj^11^	USA	Cross‐sectional case‐control	158	52.85 ± 8.26	60.12		‐	<20	
Balla^12^	Sudan	Cross‐sectional case‐control	120	53 ± 6.36	42.5	26.5667 ± 3.5606	Plasma		Euroimmun 25‐OH vitamin D ELISA kit
Dall'Angol^13^	Brazil	Cross‐sectional	114	60.47 ± 10	43	30 ± 4	Serum	41	Chemiluminescence
Dong^14^	China	Case‐control	276	56.75 ± 10.69	55	23.4802 ± 3.0819	Plasma		
El Askary^15^	Saudi Arabia	Case‐control	172	45.87 ± 9.43	55	27.6353 ± 2.2558	Serum	<20 ng/mL	Abcam human vitamin D enzyme‐linked immunosorbent assay (ELISA) kit
Felicio^16^	Brazil	Cross‐sectional	73	28.6 ± 8.1	44	25.4329 ± 5.0854	Serum	<20	Chemiluminescence immunoassay
Felicio^17^	Brazil	Cross‐sectional	1576	62.2 ± 12	37.3	29.5 ± 5	Serum	<20	DiaSorin LIAISON 25(OH)Vitamin D TOTAL chemiluminescence immunoasay
Gameil^18^	Egypt	Case‐control	294	48.45 ± 8.26	57	33.6883 ± 3.9451	Serum		
Gherdan^19^	Romania	Cross‐sectional	218	49.6 ± 8.64	59	27.25 ± 3.96	Serum	≤50	Electrochemiluminescence immunoassay
Guo^20^							Serum		
Huang^21^	China	Cross‐sectional	559	57.41 ± 14.14	59.2	24.6205 ± 3.5621	Serum		Electrochemiluminescence immunoassay
Inci^22^	Turkey	Cross‐sectional	141	61.65 ± 9.63	52.48	30.1221 ± 4.9622	Serum		Direct competitive chemiluminescence immuno‐ assay method (DiaSorin, Stillwater)
Kavuparambil^23^	India	Cross‐sectional	120	54.65 ± 5.9	52.5		Serum		
Li^24^	China	Cross‐sectional	207	56.6 ± 12.5	51.7	24.4865 ± 3.817	Serum	<15	Electrochemiluminescence immunoassay
Peng^25^	China	Cross‐sectional	448	62.5 ± 17.1	54.9	25.9321	Serum	<20	E601 modular ana‐ lyzer (Roche Diagnostics)
Senyigit^26^	Turkey	Cross‐sectional	203	56.03 ± 8.59		30.655 ± 6.4146	Serum	<9 n	HPLC method on a Roche Cobas E 601, Germany
Shao^27^	China	Cross‐sectional	502	49.95 ± 10.08	72.9		Serum		Chemiluminescence
Sonkar^28^	India	Cross‐sectional	150	50.01 ± 8.2	53.3		Serum		Chemiluminescent immunoassay technology by Roche Cobas e‐411 Immunoanalyzer.
Wang^29^	China	Cross‐sectional	4033	67.09 ± 8.65	46.2	25.0 ± 3.6	Serum		Chemiluminescence immunoassay
Xiao^30^	China	Case–control	300	58.5 ± 9.51	44.67	25.66 ± 2.831	Serum	<15	High‐pressure liquid chromatography
Xie^31^	China	Cross‐sectional	351	55.1 ± 10.35	68.9		Serum	< 30	Electrochemiluminescence immunoassay
Zhao^32^	China	Cross‐sectional	815	59.7 ± 11.4	69.93	25.2527 ± 3.5414	Serum	< 10	Electrochemiluminescence assay (Roche, Switzerland)

### 3.3 Study outcomes

#### 3.3.1 Vitamin D levels in diabetic patients

**FIGURE 2:**
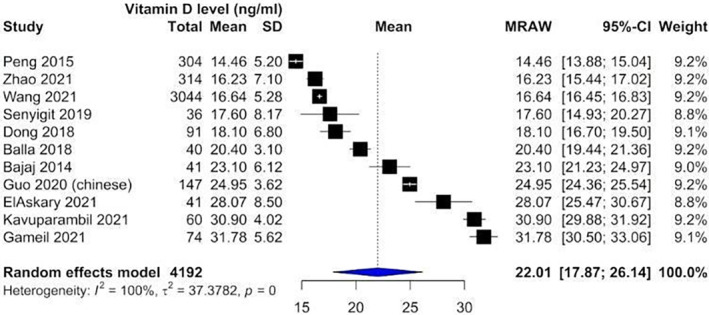
Forest plot showing vitamin D levels among diabetic patients not suffering from nephropathy.

**FIGURE 3:**
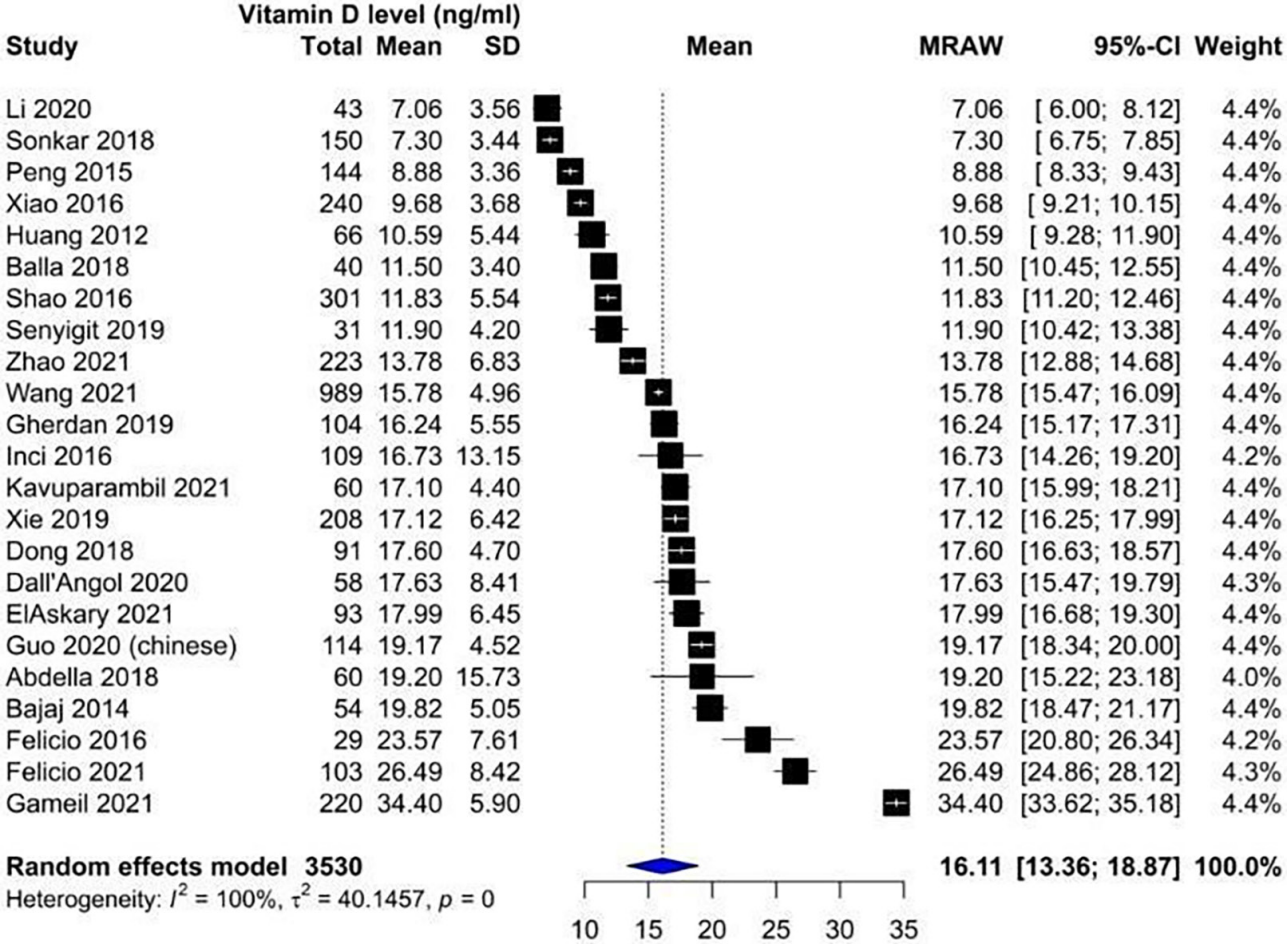
Forest plot showing vitamin D levels among diabetic patients suffering from nephropathy.

**FIGURE 4:**
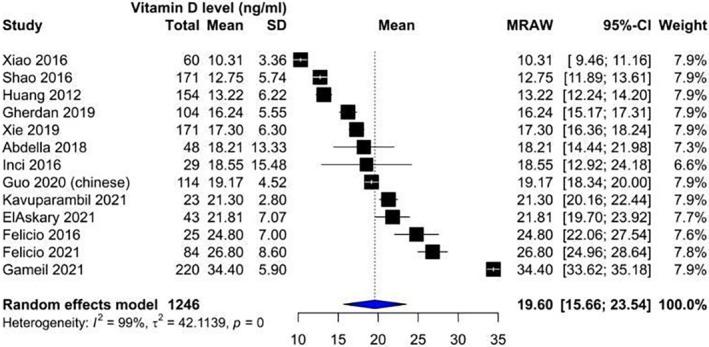
Forest plot showing vitamin D levels among diabetic patients with detected microalbuminuria.

**FIGURE 5:**
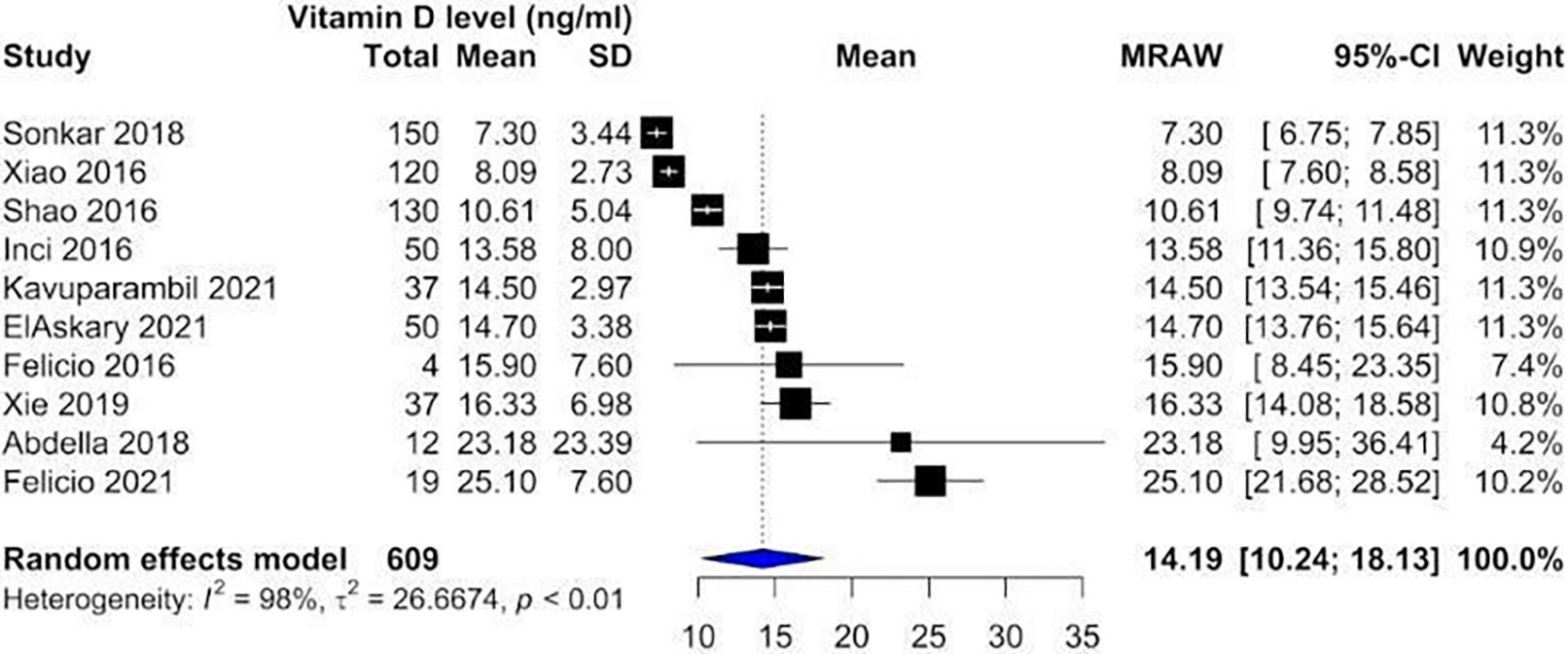
Forest plot showing vitamin D levels among diabetic patients with detected macroalbuminuria.

Our analysis showed that vitamin D levels were higher among patients without nephropathy (Figure 2: Mean 22.1, CI: 17.87–26.14) compared with patients suffering from nephropathy (Figure 3: Mean 16.11, CI: 13.36–18.87). These levels decreased as DN progressed from microalbuminuria (Figure 4: Mean 19.60, CI: 15.66–23.54) to macroalbuminuria (Figure 5: Mean 14.19, CI: 10.42–18.13). As DN further advanced, vitamin D level kept on decreasing; Grade 4 DN showed a mean level of 11.45 (CI: −1.49 to 24.40) (Figure S1), while the lowest levels were observed in Grade 5 DN (9.11, CI: 0.87–17.36) (Figure S2).

#### 3.3.2 Diabetic patients without nephropathy versus diabetic patients with nephropathy

**FIGURE 6:**
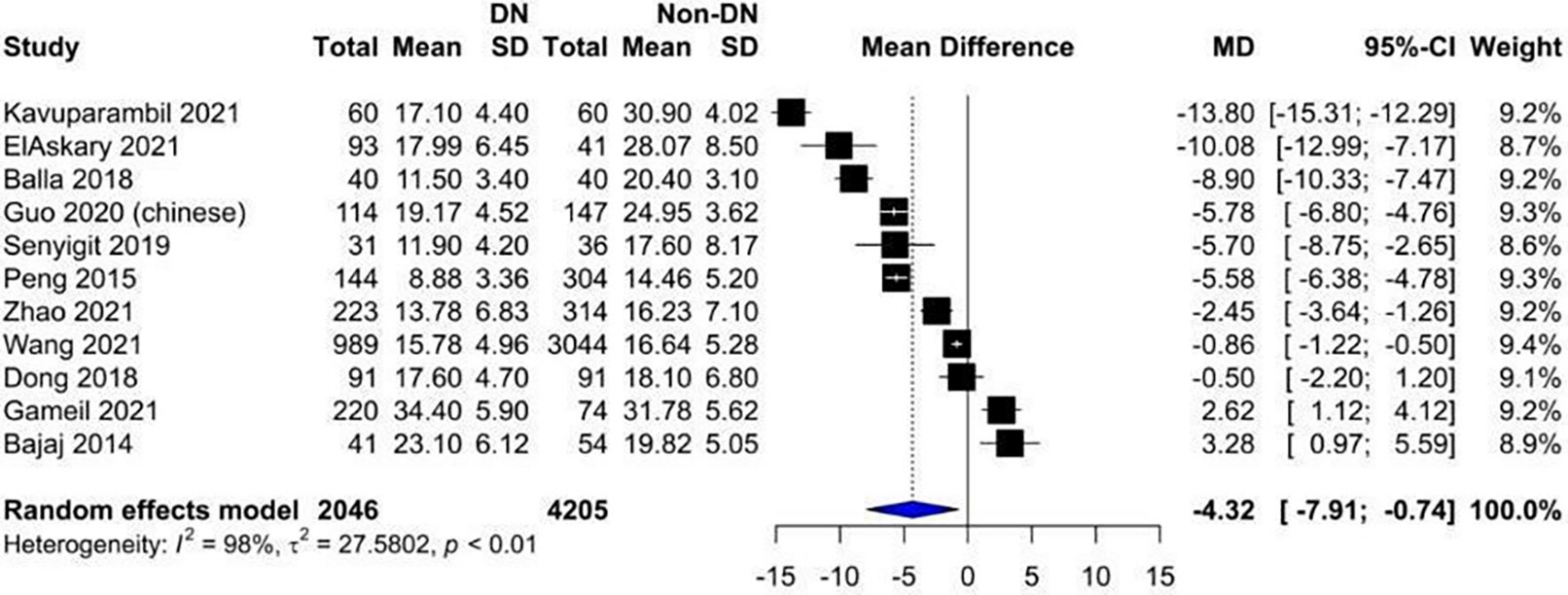
Forest plot showing the association between vitamin D levels in diabetic patients without nephropathy and diabetic patients with nephropathy.

Our analysis showed that vitamin D level was statistically significantly lower in diabetic patients with nephropathy compared with those without nephropathy (MD = ‐4.32, 95% CI [−7.91 to 0.74], *p*‐value = .0228) (Figure 6).

#### 3.3.3 Diabetic patients with microalbuminuria versus diabetic patients with normoalbuminuria

**FIGURE 7:**
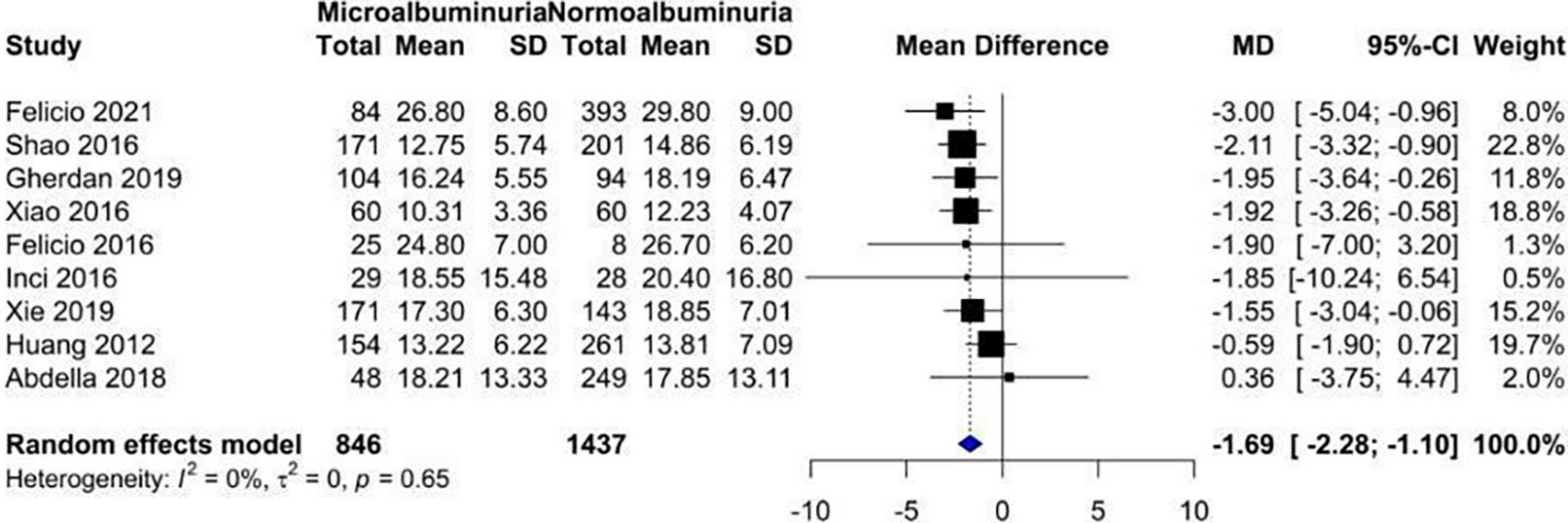
Forest plot showing the association between vitamin D levels in diabetic patients with microalbuminuria and diabetic patients with normoalbuminuria.

Our analysis showed that vitamin D level was significantly lower in diabetic patients with microalbuminuria compared with those with normoalbuminuria (MD = −1.69, 95% CI [−2.28 to −1.10] *p*‐value = .0002) (Figure 7).

#### 3.3.4 Diabetic patients with microalbuminuria versus diabetic patients with macroalbuminuria

**FIGURE 8:**
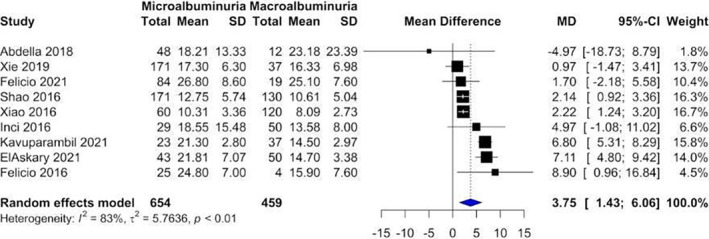
Forest plot showing the association between vitamin D levels in diabetic patients with macroalbuminuria and diabetic patients with microalbuminuria.

Our analysis showed that vitamin D level was statistically significantly lower in diabetic patients with macroalbuminuria compared with those with microalbuminuria (MD = 3.75, 95% CI [1.43–6.06], *p*‐value = .0058) (Figure 8).

#### 3.3.5 Grade 4 diabetic nephropathy versus Grade 5 diabetic nephropathy

Our analysis showed that vitamin D level was lower in Grade 5 diabetic nephropathy compared with Grade 4, but not statistically significant (MD = 2.29, 95% CI [−2.69; 7.28], *p*‐value = .1862) (Figure: S3).

### 3.4 Age and vitamin D levels

In both the age ≥55 years and age <55 years subgroups, our pooled analysis revealed no statistically significant difference between vitamin D levels in DN versus non‐DN patients (MD = −4.32, 95% CI [−88.1–0.17]); (MD = −3.59, 95% CI [84.25–77.08]), respectively. (Figure S4).

#### 3.4.1 Duration of diabetes and vitamin D levels

In both the duration of diabetes ≥10 years and age <10 years subgroups, our pooled analysis revealed no statistically significant difference between vitamin D levels in DN versus non‐DN patients (MD = −7.23, 95% CI [−28.32 to 13.87]); (MD = 1.41, 95% CI [−7.93–5.11]), respectively. (Figure S5).

## 4 DISCUSSION

Our analysis revealed that vitamin D was significantly lower in DN patients than in diabetics without nephropathy. On comparing diabetic patients with normoalbuminuria, microalbuminuria, and macroalbuminuria, we found that vitamin D levels decreased as the disease progressed. Notwithstanding, we detected an insignificant association between Grade 4 and Grade 5 DN. Age and the duration of diabetes did not significantly impact vitamin D levels.

Multiple studies have shown that serum levels of vitamin D were lower among DN patients; a study conducted in China by Li et al.^34^ showed that DN patients had a high prevalence of vitamin D deficiency (<20 ng/mL) and proteinuria was higher among these patients. Similar findings were demonstrated among DN patients in the US by Diaz et al.^35^ in their cross‐sectional and in Spain by Sanchez‐Hernandez et al.^36^

Our analysis showed that vitamin D progressively declined as the albuminuria advanced from normoalbuminuria to micro and macroalbuminuria, which aligns with the findings of Hong et al.^37^ who concluded that serum vitamin D levels negatively correlated with the urinary albumin‐to‐creatinine ratio (UACR). However, they detected a positive correlation between vitamin D levels and the age of diabetic patients. This contradicts our findings as our analysis showed an insignificant association between the age of the diabetics and vitamin D levels. Another study conducted by Kondo et al. on Japanese patients showed that the prevalence of vitamin D deficiency among DN was low; nevertheless, albuminuria was associated with low serum vitamin D levels.^38^

In a previous meta‐analysis by Derakhshanian et al.^39^ serum vitamin D levels were inversely related to the risk of developing DN, reinforcing our findings, which showed that DN patients had lower serum vitamin D levels than non‐DN patients. The prior meta‐analysis included six studies and 3700 patients in their observational arm, while we included 23 studies and 7722 patients. A recent meta‐analysis by Yammine et al.^40^ concluded that DN patients suffer from an increased prevalence of vitamin D deficiency compared with diabetics without DN. However, their study did not analyse the different grades of DN and its relation with serum vitamin D levels. The current study analysed the progression of DN, the degree of albuminuria, and vitamin D levels. This enabled us to draw a crucial conclusion about the potential use of vitamin D as a prognostic marker in DN patients. Our results showed that as DN progressed, patients' vitamin D levels kept declining, with patients suffering from macroalbuminuria having the lowest vitamin D levels compared with DN suffering from microalbuminuria or normoalbuminuria. Since lower vitamin D levels were associated with more severe forms of DN, serum vitamin D levels could serve as a prognostic marker for DN. A similar concept was demonstrated by Schiller et al.^41^ where end‐stage renal disease (ESRD) diabetic patients had a higher prevalence of vitamin D deficiency, and this deficiency was associated with increased all‐cause mortality in these patients. Moreover, Zomorodian et al.^42^ considered serum vitamin D levels of 21 ng/mL or less as a cut‐off point for having microalbuminuria among DN patients; this agrees with our results, as patients with macroalbuminuria had vitamin D levels of 14.19 ng/dL. It is important to note that, according to our analysis, once the diabetic patients reached the stage of macroalbuminuria, serum vitamin D levels failed to discriminate between Grade 4 and Grade 5 DN.

Multiple clinical trials have recently explored the potential benefits of vitamin D supplements in treating or preventing DN. A double‐blinded clinical trial by Momeni et al.^43^ concluded that vitamin D supplements decreased proteinuria among DN patients. Similar findings were seen in the clinical trial conducted by Esfandiari et al.^44^ On the other hand, Derakhshanian et al.^39^ in their meta‐analysis, demonstrated that vitamin D supplementation did not decrease UACR. A recent meta‐analysis conducted by Xuan et al. showed that vitamin D supplementation among DN patients was significantly associated with a reduction in proteinuria.^45^ Nevertheless, multiple low quality papers were included in their analysis and their sample size was limited to 651 patients, rendering more trials necessary to explore the potential benefit of supplementation.

### 4.1 Strengths and limitation

Our analysis included 23 studies originating from 10 different countries and 5 continents. That enabled us to increase the generalizability of findings to apply our results globally. Furthermore, we included different grades and degrees of albuminuria among DN patients to assess early and advanced disease stages. Lastly, we analysed the mean vitamin D levels in each study instead of the prevalence of vitamin D deficiency among DN as studies had different cut‐off values for vitamin D deficiency. This allowed us to draw more accurate conclusions.

Some included studies did not specify whether the patients suffered from Type 1 or Type 2 DM. Subsequently, we could not run a separate analysis on each type. Two out of the 23 included studies used plasma samples instead of serum; these studies account for 1.6% of the total sample size (Table 1). There was insufficient data to perform a subgroup analysis based on the sample type. Some studies have shown that sample type causes a significant difference in the measured vitamin D concentration, while others did not.^46^, ^47^

Lastly, we lacked data regarding the survival of vitamin D deficient DN patients; more studies are warranted regarding the prognostic value of vitamin D deficiency.

## 5 CONCLUSION

Vitamin D levels are lower among DN patients and keep declining as the disease progresses, suggesting its potential benefit as a prognostic marker. However, on reaching the macroalbuminuria stage (Grade 4 and Grade 5), vitamin D is no longer a discriminating factor.

## AUTHOR CONTRIBUTIONS

**Yomna E. Dean:** Conceptualization (equal); data curation (lead); investigation (lead); methodology (lead); project administration (lead); supervision (equal); validation (equal); visualization (equal); writing – original draft (lead); writing – review and editing (lead). **Sameh Samir Elawady:** Conceptualization (lead); formal analysis (lead); software (equal); writing – original draft (equal). **Wangpan Shi:** Data curation (equal); writing – original draft (equal). **Ahmed A. Salem:** Data curation; writing – original draft. **Arinnan Chotwatanapong:** Data curation (equal); writing – original draft (equal). **Haya Ashraf:** Data curation (equal); writing – original draft (equal). **Tharun Reddi:** Data curation (equal); writing – original draft (equal). **Prashant Obed Reddy Dundi:** Writing – review and editing (equal). **Waleed Yasser Habash:** Data curation (equal); writing – original draft (equal). **Mohamed Yasser Habash:** Data curation (equal); writing – original draft (equal). **Safaa Ahmed:** Data curation (equal); writing – original draft (equal). **Hana M. Samir:** Data curation (equal); writing – original draft (equal). **Ahmed Elsayed:** Data curation (equal). **Aryan Arora:** Writing – review and editing (equal). **Abhinav Arora:** Writing – review and editing (equal). **Abdelrahman Elsayed:** Data curation (equal). **Tamer Mady:** Writing – review and editing (equal). **Yousef Tanas:** Data curation (equal). **Yusef Hazimeh:** Supervision (equal). **Mohamed Alazmy:** Writing – review and editing (equal). **Hani Aiash:** Supervision (lead); visualization (lead); writing – review and editing (lead).

## CONFLICT OF INTEREST STATEMENT

Authors have no conflicts of interest to declare.

## Supporting information


Figure S1.

Table S1.

Table S2.

## Data Availability

All data is available upon request to the corresponding author.
