# Supplementary material for: Progression of diabetic nephropathy and vitamin D serum levels: A pooled analysis of 7722 patients
Source: Endocrinol Diabetes Metab. 2023 Sep 24;6(6):e453. doi: 10.1002/edm2.453 (PMC10638614; doi:10.1002/edm2.453)

**Supplementary Figures:**

Figure S1: Forest Plot showing Vitamin D levels among diabetic patients suffering from grade 4 diabetic nephropathy.


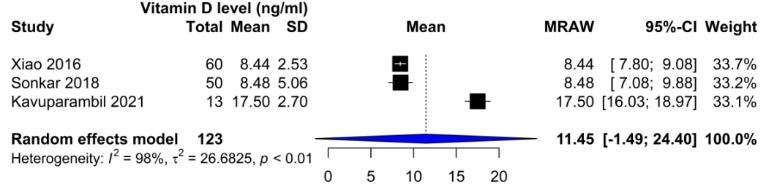


Figure S2: Forest Plot showing Vitamin D levels among diabetic patients suffering from grade 5 diabetic nephropathy.


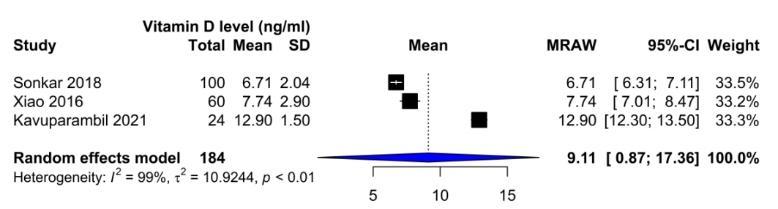


Figure S3: Forest plot showing the association between vitamin D levels in grade 4 and grade 5 diabetic nephropathy patients.


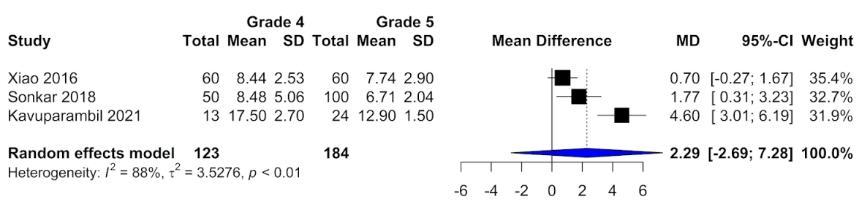


Figure S4: Forest plot showing the association between age, vitamin D levels, patients suffering from diabetic nephropathy, and diabetic patients not suffering from nephropathy.


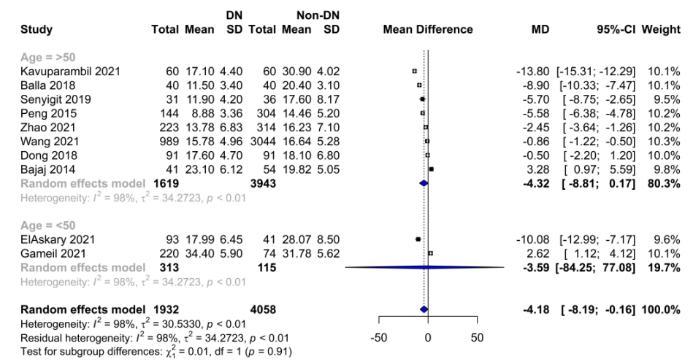


Figure S5: Forest plot showing the association between diabetes duration, vitamin D levels, patients suffering from diabetic nephropathy, and diabetic patients not suffering from nephropathy.


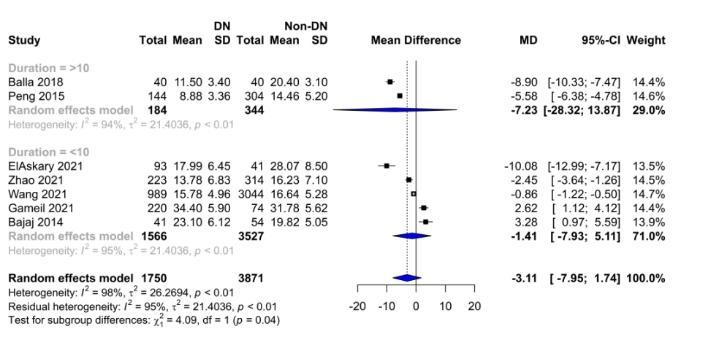

Supplement: Supplementary file 1 — Figure S1. [file EDM2-6-e453-s001.docx]
