# Supplementary material for: Progression of diabetic nephropathy and vitamin D serum levels: A pooled analysis of 7722 patients
Source: Endocrinol Diabetes Metab. 2023 Sep 24;6(6):e453. doi: 10.1002/edm2.453 (PMC10638614; doi:10.1002/edm2.453)
Supplement: Supplementary file 2 — Table S1. [file EDM2-6-e453-s002.docx]

Table S1: Quality assessment of case control studies

| Author Year | Selection | | | | Comparability | Exposure | | | Total |
| --- | --- | --- | --- | --- | --- | --- | --- | --- | --- |
|  | 1) Is the Case Definition Adequate? | 2) Representativeness of the Cases | 3) Selection of Controls | 4) Definition of Controls | 1) Comparability of Cases and Controls on the Basis of the Design or Analysis | 1) Ascertainment of exposure | 2) Same method of ascertainment for cases and controls | 3) Non-Response rate |  |
| Peng 2015 | 1 | 1 | 0 | 1 | 2 | 1 | 1 | 1 | 8 |
| Dong 2018 | 1 | 1 | 1 | 1 | 0 | 1 | 1 | 1 | 7 |
| ElAskary 2021 | 1 | 1 | 0 | 1 | 0 | 1 | 1 | 1 | 6 |
| Gameil 2021 | 1 | 1 | 0 | 1 | 1 | 1 | 1 | 1 | 7 |
| Bajaj 2014 | 0 | 0 | 1 | 1 | 1 | 0 | 1 | 0 | 4 |
