# Supplementary material for: Progression of diabetic nephropathy and vitamin D serum levels: A pooled analysis of 7722 patients
Source: Endocrinol Diabetes Metab. 2023 Sep 24;6(6):e453. doi: 10.1002/edm2.453 (PMC10638614; doi:10.1002/edm2.453)
Supplement: Supplementary file 3 — Table S2. [file EDM2-6-e453-s003.docx]

Table S2: Quality Assessment of cross-sectional studies

| Author Year | Selection | | | | | | | | | Comparability | | Exposure | | | | Total |
| --- | --- | --- | --- | --- | --- | --- | --- | --- | --- | --- | --- | --- | --- | --- | --- | --- |
|  | 1) Representativeness of the exposed cohort | | 2) Sample size | | | 3) Ascertainment of exposure | | 4) Non-respondents | | 1) Comparability of cohorts on the basis of the design or analysis | | 1) Assessment of outcome | | 2) Statistical test | |  |
| Li 2020 | | 1 | | 1 | 1 | | 0 | | 1 | | 1 | | 1 | | 6 | |
| Zhao 2021 | | 1 | | 1 | 2 | | 1 | | 1 | | 1 | | 1 | | 8 | |
| Wang 2021 | | 1 | | 1 | 2 | | 1 | | 2 | | 1 | | 1 | | 9 | |
| Kavuparambil 2021 | | 0 | | 0 | 1 | | 1 | | 1 | | 1 | | 1 | | 5 | |
| Senyigit 2019 | | 0 | | 0 | 1 | | 1 | | 1 | | 1 | | 1 | | 5 | |
| Balla 2018 | | 0 | | 0 | 1 | | 1 | | 1 | | 1 | | 1 | | 5 | |
| Shao 2017 | | 0 | | 1 | 2 | | 1 | | 1 | | 1 | | 1 | | 7 | |
| Abdella 2018 | | 1 | | 1 | 1 | | 1 | | 1 | | 1 | | 1 | | 7 | |
| Gherdan 2019 | | 0 | | 1 | 1 | | 0 | | 1 | | 1 | | 1 | | 5 | |
| Dall'Agnol 2020 | | 0 | | 0 | 2 | | 0 | | 1 | | 1 | | 1 | | 5 | |
| Xie 2019 | | 0 | | 1 | 2 | | 1 | | 1 | | 1 | | 1 | | 7 | |
| Inci 2016 | | 0 | | 0 | 2 | | 1 | | 1 | | 1 | | 1 | | 6 | |
| Felicio 2016 | | 0 | | 0 | 2 | | 1 | | 1 | | 1 | | 1 | | 6 | |
| Felicio 2021 | | 1 | | 1 | 2 | | 1 | | 2 | | 1 | | 1 | | 9 | |
| Sonkar 2018 | | 1 | | 1 | 1 | | 0 | | 0 | | 1 | | 1 | | 5 | |
| Huang 2012 | | 0 | | 1 | 1 | | 1 | | 2 | | 1 | | 1 | | 7 | |
